# Fungal Community Structural and Microbial Functional Pattern Changes After Soil Amendments by Oilseed Meals of *Jatropha curcas* and *Camelina sativa*: A Microcosm Study

**DOI:** 10.3389/fmicb.2019.00537

**Published:** 2019-03-29

**Authors:** Ping Hu, Liangjun Wu, Emily B. Hollister, Autumn S. Wang, Anilkumar C. Somenahally, Frank M. Hons, Terry J. Gentry

**Affiliations:** ^1^Key Laboratory of Environment and Health (HUST), Ministry of Education and Ministry of Environmental Protection, State Key Laboratory of Environmental Health (Incubation), School of Public Health, Tongji Medical College, Huazhong University of Science and Technology, Wuhan, China; ^2^Department of Forensic Medicine, Tongji Medical College, Huazhong University of Science and Technology, Wuhan, China; ^3^Baylor College of Medicine, Houston, TX, United States; ^4^Texas Children’s Hospital, Houston, TX, United States; ^5^Department of Soil and Crop Sciences, Texas A&M University, College Station, TX, United States

**Keywords:** oilseed meal, *Jatropha curcas*, *Camelina sativa*, microbial communities, Biolog, pyrosequencing

## Abstract

The meals after oil extraction from many oilseed crops have nutrition and biofumigation potential for land application. Oilseed meal (SM) from the dedicated bioenergy crop *Jatropha curcas* were implicated to contain compounds that have antibacterial properties on some soil pathogens. However, little is known about its effect on non-targeted soil microbial community, especially on fungi. SM from *Camelina sativa* contains moderate level of glucosinolates (GLS) and was under studied. To investigate soil fungal community responses to jatropha and camelina SMs, we conducted a lab based microcosm study, amending soil with 1% SMs of jatropha, camelina, flax, and biomass of wheat straw. Fungal community abundance and structure were analyzed based on the ITS region using qPCR and tag-pyrosequencing. Microbial functional changes were examined by community level physiological profile (CLPP) using Biolog assay. Both SMs from jatropha and camelina showed biofumigant properties and inhibited fungal proliferation. Jatropha SM significantly altered soil fungal community structures with lower fungal biodiversity and higher *Chaetomium* composition. Camelina SM amended soil promoted *Fusarium* proliferation. CLPP indicated sequential hierarchy for C metabolism in the oilseed-amended microcosms was generally complex C > phosphate-associated C > carboxylic acids > carbohydrates > amines > amino acids. No significant difference in CLPP was detected due to the type of SM treatment. Our data indicate that both SMs of jatropha and camelina have biofumigant properties and can differentially impact soil microbial communities, and the changes were relatively persistent over time. Microbial functional patterns on the other side were not impacted by SM type. Our study revealed biofumigant and nutritional influence of SMs from dedicated biofuel plants on soil microbial community. This information will help properly using jatropha and camelina SMs for pathogen control while minimizing their negative impacts on non-target microorganisms. However, further studies in the field are demanded to investigate their influences in real practice.

## Introduction

Extraction of oil from oilseed crops and subsequently transforming the oil into biodiesel is one of the major pathways for the production of biofuels. With increased interest in the use of biofuels to supplement fossil fuel supplies, increasing amounts of oilseed meals (SMs), which are the by-products (residual) remaining after the oil extraction process, will be produced. One of the possible uses for these SMs includes land application as soil organic amendments, due to their high nutrient contents (C, N, P etc.). Another related, potential application for some SMs (i.e., those containing allelochemicals) is in biofumigation strategies to control pathogens, insects, and/or weeds (Hu et al., 2011). Although there has been a relatively large amount of research on using SMs or plant materials as organic fertilizers and for controlling pathogens and weeds (Mazzola et al., 2007; Moore et al., 2010; Snyder et al., 2010; Wang et al., 2012), relatively limited research has been focused on dedicated bioenergy oilseed crops such as jatropha. In addition, very limited information is available about the impact of these SMs on the soil microbial community, despite the critical importance of the soil microorganisms to many soil processes, plant health, and soil quality.

Many SMs have a potential role in biofumigation and have been demonstrated to suppress several plant pathogens including fungi and oomycetes such as *Phymatotrichopsis omnivora* (Duggar) Hennebert (Hu et al., 2011), *Aphanomyces euteiches* f. sp. *pisi* (Smolinska et al., 1997), *Rhizoctonia* spp. (Cohen et al., 2005), *Pratylenchus penetrans* and *Pythium* spp. (Mazzola et al., 2014), and *Fusarium* wilt (Ren et al., 2018). Such studies using SMs are still relatively limited although meals from the oilseed crop seeds (where the allelochemicals tend to be more concentrated) have been demonstrated to be more efficient and effective as a biofumigant than other plant tissues (Mazzola et al., 2001; Mazzola and Zhao, 2010). The mechanisms for pathogen control by SMs have been attributed to either the allelochemicals released or/and plant systemic resistance conferred by changes in the soil microbial community (Cohen and Mazzola, 2006; Hu et al., 2011). Other possible nutritional consequences included an unfavorable increase in acidity and increases in populations of antagonistic microorganisms (Hine et al., 1979). In addition to a potential impact on plant pathogens, changes in soil microbial communities may impact other ecosystem processes. S Numerous studies have demonstrated that the addition of organic amendments can alter, at least transiently, soil microbial communities with these impacts possibly being either beneficial or detrimental to soil quality and pathogen control (Hamel et al., 2005; Yao et al., 2006; Lejon et al., 2007; Omirou et al., 2011; Yim et al., 2017). In addition, studies have shown that isothiocyanates (ITCs), such as those produced by many *Brassica* spp., can decrease soil microbial populations and specifically nitrifying bacteria (Bending and Lincoln, 1999; Rumberger and Marschner, 2003).

Jatropha plant tissue and meals from the oilseed kernels contains active antibacterial and antioxidant compounds including phorbol ester, phenolics, flavonoids, saponins, diterpenoids, 3,4,3’-Tri-*O*-methylellagic acid, and 1-*O*-Linoleoyl-glycerol (Devappa and Makkar, 2011; Oskoueian et al., 2011; Okoh et al., 2016; Ha et al., 2018; Wang et al., 2018), which made it problematic as a potential feedstock for animals, but on the other hand could be promising as an antibiotic candidate. Detoxification of jatropha oilseed meals through bioaugmented composting or fermentation can be effective (Chaturvedi et al., 2013; Wang et al., 2013) but requires extra cost and processes. Breeding non-toxic variety or generating transgenic strains with less phorbol ester could also be successful (Li et al., 2016), but was limited in real practice by farmers due to lack of understanding on how to grow this plant (Ha et al., 2018). Some studies showed that land application of jatropha SM as organic fertilizer is a safe practice to consumers and to the environment, and can effectively support plant growth and increase soil organic C, microbial biomass and activities (Srinophakun et al., 2011; Anand et al., 2015; Elbl et al., 2016). The toxic phorbol ester released from jatropha SM was not detected in the plant and degraded to negligible level in the soil quickly (Srinophakun et al., 2011; Anand et al., 2015). Since the antibacterial properties of jatropha kernel meals have been recognized, it is promising to use jatropha SMs as biofumigant for pathogen control, although only limited researches demonstrating the bioactive effects of jatropha SMs on targeted and non-targeted microorganisms when applied into soil (Chaudhary et al., 2011; Hu et al., 2011). Chaudhary et al. (2011) found jatropha SM amendment to soil changed soil microbial community and promoted microbial diversity and activity according to FAME and enzyme assay analysis, suggesting jatropha SM a favorable amendment for soils but requires further investigation using techniques such as high throughput sequencing with better resolution to specify which microorganisms were affected.

In the past, most of microbial community studies have been focused upon the soil bacterial community (Lauber et al., 2009), even though the soil fungal community is likely to be of as much, or even greater, importance than bacteria to many processes such as organic matter formation and decomposition (Bååth and Anderson, 2003; Boer et al., 2010; Lindahl et al., 2010; Baldrian et al., 2012). Nevertheless, such studies on soil fungal communities have been quite limited primarily because it has been difficult to describe most fungal species, estimate their diversity, distinguish individual taxa, and understand the ecological roles that fungi played (Hawksworth, 2001; Bailey et al., 2002; McGuire and Treseder, 2010). Moreover, the handful of studies that have investigated the impacts of SMs on soil fungal composition have used low resolution techniques such as fatty acid methyl ester analysis which provided information regarding community shifts but little-to-no information regarding which specific organisms were being impacted (Wang et al., 2012). More recent studies have revealed soil fungal community changes due to SM amendments using higher resolution techniques including pyrosequencing (Hollister et al., 2013; Mazzola et al., 2014; Hu et al., 2015; Ren et al., 2018; Siebers et al., 2018), although they all focused on brassicaceous or ITC amended SMs that produce high levels of ITCs. To date, no study investigated detailed soil microbial community responses to amendments of the dedicated bioenergy oilseed crop *Jatropha curcas* SM that contains non-glucosinolates biocidal chemicals. Besides, only limited research investigated applying SMs containing lower level of GLS (e.g., camellia SM) (Hu et al., 2011), and found no fumigant property on targeted pathogen (Ren et al., 2018). Moreover, to explore the microbial communities through pyrosequencing can only obtain structural composition, while soil functional information after SM treatments including microbial community level physiological profile and soil C mineralization dynamics is also very important, although a recent study reported that several brassicacous SMs did not lead to soil biodiversity changes according to Biolog data (Scotti et al., 2018).

The objective of this study is to investigate the effects of SMs on soil microbiota, with a focus on dedicated bioenergy oilseed crop *Jatropha curcas* and its influence on soil fungal community. We will integrate information from soil fungal absolute abundance, phylogenetic structure, and functional profiles to elucidate soil fungal community responses due to SM incorporation.

## Materials and Methods

### Soil Collection and Characterization

We used Weswood loam (fine-silty, mixed, superactive, thermic, Udifluventic Haplustept) in this study. It is an alluvial soil in the flood plain of the Brazos River in south central Texas. Selected characteristics can be found in a previous study (Wang et al., 2012). This type of soil is well drained and has been used as irrigated cropland (Soil Survey Staff, Natural Resources Conservation Service and United States Department of Agriculture et al., 2008). Bulk soil were collected from 0–15 cm depth and transported to lab in 40L plastic buckets in May 2008. Soil samples were then homogenized and air-dried. Water was added to field moisture level at the beginning of incubation.

### SM Analysis

*Triticum aestivum* L.(wheat) straw was from the Texas AgriLife Research Farm near College Station, TX, United States. Information on the source and analysis on oilseed meals of *J. curcas* L. (jatropha), *Camelina sativa* (L.) Crantz (camelina) and *Linum usitatissimum* L. (flax) used in our study can be obtained from a previous study (Hu et al., 2011). Briefly, all oilseed meals were obtained by processing seeds with a Komet Oil Press, ground with mortar and pestle and passed through 1mm sieve. Total C and N were determined by a high-temperature combustion process using an Elementar Vario Max CN analyzer. Plant B, Ca, Cu, Fe, K, Mg, Na, P, S, and Zn were determined using a nitric acid digestion and ICP analysis.

### Experimental Plan

Each microcosm contained 400 g dry soil in a 1-L glass jar, and organic amendments of SMs and wheat straw were incorporated into soils at an application rate of 1.0 (w/w), or a field-equivalent of approximately 18 Mg ha^-1^. The microcosms were maintained at 13% (w/w) water content (approximately 40% field capacity) and incubated at 25°C, which is approximately an average temperature (29.4°C) of south Texas in summer under aerobic conditions. Each treatment had 3 replicated microcosms, with a series of unamended controls receiving no organic addition. In total, 15 microcosms were set up with 5 treatments including unamended control. To track potential changes in community level physiological profiling with Biolog Ecoplates, 1 g of soil from each microcosm was subsampled and processed at days 3, 7, 14, 28, 77, and 133. Another 5 g of sub samples were collected at days 3, 7, 14, 21, 28, and 77 to investigate temporal changes in soil bacterial and fungal abundance using quantitative real time polymerase chain reaction (qPCR). These soil sub-samples at days 3, 21, and 77 were used in soil fungal community analysis with pyrosequencing. Soil sub-samples for qPCR and pyrosequencing were stored at -80°C until DNA extraction.

### Functional Diversity Using Biolog Analysis

Soil CLPP based on carbon (C) source utilization patterns was obtained using Biolog EcoPlates (Hayward, CA, United States) containing 31 different C sources. To carry out the procedure, 1 g of wet soil was collected from each sample and suspended in 9 mL of 0.87% saline solution (8.7 g NaCl/L w/v), and then diluted 100 times further. Aliquots of 150 μL of the resulting soil solution were injected in EcoPlate wells with a multichannel pipette and incubated at 25°C in the dark for at least 96 h, during which absorbance was measured at 590 nm every 24 h using an ELx808 Microplate Reader (Biolog, Inc., Hayward, CA, United States). The average well-color development (AWCD) was calculated from each plate at each time point. For each plate, those time points of readings that had an AWCD closest to 0.75 were selected for data analysis (Garland, 1996) and normalized dividing by the AWCD to reduce biases due to different inoculum densities (Garland, 1997). For data analysis, the 31 C sources were grouped into 6 categories including carboxylic acids, carbohydrates, complex C sources, phosphate-associated C, amino acids, and amines (Supplementary Table S1).

### DNA Extraction and Quantification

Community DNA was extracted from 0.5 g soil samples using a PowerSoil DNA extraction kit (Mo Bio Laboratories, Inc., Carlsbad, CA, United States). Extracted DNA was purified with illustra MicroSpin S-400 HR columns (GE Healthcare Bio-Sciences Corp, Piscataway, NJ, United States), and quantified with a NanoDrop ND-1000 spectrophotometer (NanoDrop Technologies, Wilmington, DE, United States) and Quant-iT PicoGreen dsDNA assay kit (Invitrogen Corp., Carlsbad, CA, United States). Data generated from the latter was used in the analysis.

### qPCR on General Bacterial and Fungal Abundance

Community qPCR assays based on Fierer et al. (2005; Noah et al., 2005) and Boyle et al. (2008) were used to evaluate relative abundances of soil general bacterial and fungal populations in each sample. Assays were performed in triplicate using a Rotor-Gene 6000 series thermal cycler (Qiagen, Valencia, CA, United States). Each 10 μL reaction for qPCR contained: 4.5 μL 2.5x RealMasterMix with 20x SYBR solution (5Prime, Inc., Gaithersburg, MD, United States), 1.0 μL BSA (10 mg mL^-1^), 0.5 μL of each primer (10 μM,), 2.5 μL molecular-grade water, and 1.0 μL template DNA (2.5 ng μL^-1^). Thermocycling consisted of an initial denaturation at 95°C for 15 min, followed by 40 cycles of 95°C for 1 min and annealing temperature at 53°C for 30 s, and 72°C for 1 min. Primer sets of Eub338/518 (Noah et al., 2005) and 5.8S/ITS1F (Boyle et al., 2008) were used for bacteria and fungi, respectively. Plasmid standards for the bacterial and fungal relative abundance by qPCR were generated as described by Somenahally et al. (2011). Values representing the mean of three biological replicates for each treatment were used to create the graphs on soil fungal and bacterial abundance.

### Fungal Tag-Encoded Amplicon Pyrosequencing and Analysis

Purified community DNA samples from all treatments including three biological replicates each were submitted to the Research and Testing Laboratory (Lubbock, TX, United States) for tag-pyrosequencing using 454 GS FLX titanium technology (454 Life Sciences, Branford, CT, United States). The fungal ITS region was amplified using primers ITS1F and ITS4 for the initial generation of the amplicons (Amend et al., 2010), and fungal amplicons were sequenced in the forward direction, generating reads from ITS1F.

All sequences were preprocessed in MOTHUR v.1.20.0 (Schloss et al., 2009). A detailed description on sequence processing can be obtained from Hu et al. (2015). We used CD-HIT-EST for clustering sequences into OTUs. Identities for representative OTUs were determined using UNITE database’s 454 pipelines. Hits with BLAST scores ≤ 200 or query percentage of alignment ≤ 60% were considered to represent unknown or unclassified fungi. The top unclassified representative sequence was then searched though NCBI BLASTn database and was identified as *Chaetomium* (accession number GU055594.1) with 100% query coverage and e value of 7e-151. The three biological replicates for each treatment were grouped for calculations on Theta-YC (Yue and Clayton, 2005) similarity metrics, and neighbor-joining tree based on Theta-YC values in MOTHUR v.1.20.0 (Schloss et al., 2009). The most abundant OTUs (top 200), that represented the majority (97%) of all the sequences produced, were selected for community taxonomic composition descriptions at genus level.

Community OTU network analysis was conducted in Cytoscape 2.8.3 using embedded spring algorithm. Nodes represent OTUs and act as objects that repel each other, while edges represent shared OTUs and act as springs that connect OTUs to each sample with a spring tension representing the abundance of shared OTUs. The nodes were arranged in the network in the way that has the lowest total tension. Samples from the same type of SM across all time points has been assigned the same color representing impact of SMs on soil fungal communities.

We “re-sampled” our fungal sequence libraries by using sub.sample function in MOTHUR resulting in randomly selected sequences from each library with equally sized sequence numbers of 1340 to compromise for sample size bias from a lot of biological diversity and richness estimators. Only the community diversity indices and richness estimators were calculated based on these reduced sized libraries. A detailed description can be found in a previous study (Hu et al., 2015).

All tag pyrosequence data from this study were deposited and made public accessible in Genbank Sequence Read Archive (SRA) under submission number of SRR8402464. The descriptions of this study were deposited under study ID of PRJNA513249, sample ID of SAMN10699230, and experiment ID of SRX5211720.

### Statistical Analysis

Variation in community qPCR values and fungal community composition at the genus level among amendment types and over time were assessed using SAS version 9.2. Proc GLM was used to test individual treatment significance. Pair-wise treatment mean comparisons were made using Least Significance Difference (LSD) when treatment was shown to be significant. Unless otherwise indicated, all statistical significance levels were set as *P* ≤ 0.05. Values of qPCR were log-transformed, and fungal genus composition values were arcsin transformed prior to analysis.

Non-metric multidimensional scaling (NMDS) of the fungal communities based upon OTU composition was carried out using the Bray–Curtis similarity metric in the PAST (Paleontological Statistics, University of Oslo) software package, version 2.08. Data are presented using the means of biological replicates for each treatment with error bars represented for standard deviation among the three replications. ANOSIM was performed to test statistical significance regarding amendment type and sampling time. PERMANOVA was performed to test statistical significance among treatments within each time point. Data from Biolog EcoPlate^TM^ analysis were subject to principal component analysis (PCA). Ordinations of replicate-level CLPP values for each treatment at different time points were performed for the first two principal components which loaded most of the variance of original data. A PCA biplot was created to show how each category of C source contributed to the separation among treatments. PERMANOVA was performed to test statistical significance among treatments within each time point.

Heatmaps were used to show the relative abundances of fungal genera for each amendment type and time point. To create the graph, values of the mean across three biological replicates for each treatment were used with heatmap function included in the gplots package for R version 2.13.0. The colored rectangles for each taxonomic group represented sequence abundances relative to the mean of all samples. All treatments were clustered with Euclidian distance-based hierarchical agglomerative clustering.

## Results

### SM and Wheat Straw Chemical Composition

The chemical compositions of the SMs, as previously determined (Hu et al., 2011) and wheat straw used in this experiment are summarized in Supplementary Table S2. All SMs generally had very high concentrations of C, N, P, and K. The C:N ratios of SMs ranged from 7.4 for camelina to 13.6 for jatropha. The brassicaceous SM (camelina) had higher concentrations of P, S, and Zn than did the other SMs. Greater Ca, however, was detected in jatropha SM. Other elemental concentrations such as Na, Mg, Cu, Fe, Mn, and B were comparable among different SMs. Wheat straw had a different chemical composition profile than the SMs. It had similar levels of C and K, but much lower amounts of N and P than the SMs did. The C:N ratio of the wheat straw was 32, which was much higher than that of the SMs. Other elemental contents such as Ca, S, Mg, Zn, Cu, and B in wheat straw were also lower, but Fe concentration was much higher (3–6 times more) than the selected SMs.

### Abundance of Soil Bacterial and Fungal Populations

Oilseed meals and wheat straw were quickly mineralized only 3 days after incorporated into soil (Supplementary Figure S1). Oilseed meal amended soil had relatively higher C mineralization rates compared with wheat straw treatment and the unamended control at earlier incubation stages (days 3 and 7). After the initial high rates, the trend dropped dramatically and stabilized at low levels, which are similar as the unamended control.

**FIGURE 1:**
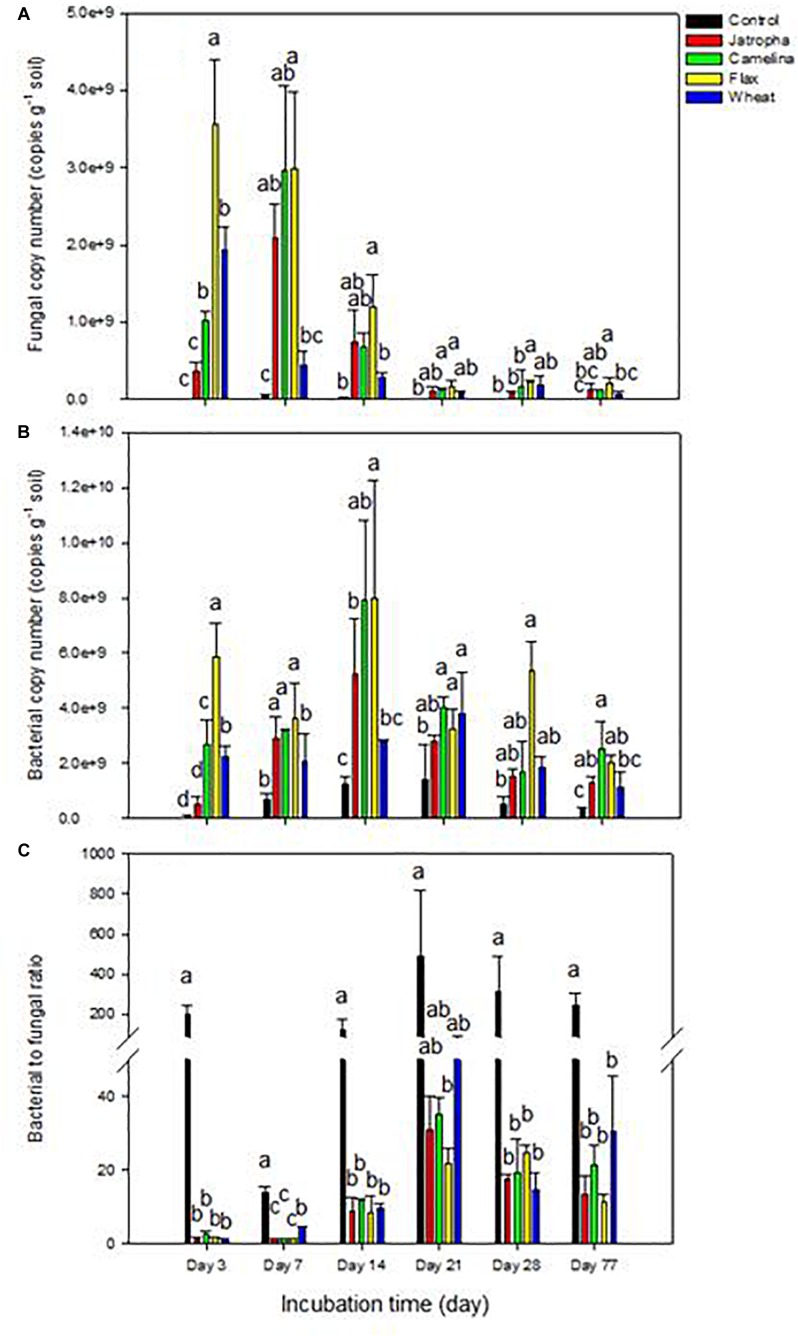
Microbial abundance by qPCR in 1% (w/w) organic material (SMs of jatropha, camelina, flax, and wheat straw) treated Weswood loam soil after 3, 7, 14, 21, 28, and 77 days of incubation at 25°C. **(A)** Soil fungal copy number. **(B)** Soil bacterial copy number. **(C)** The ratio of soil bacterial to fungal copy number. Different letters indicate significant difference at *P* < 0.05 within each day. Bars represent the means of three biological replicates for each treatment, and error bars represent the standard deviation among biological replicates.

Within 7 days, SM application enhanced soil fungal abundance dramatically (∼40-fold) compared with the unamended control (Figure 1A). Soil fungal abundance dropped significantly at day 14 and then stabilized maintaining a relatively lower level after day 21 for all treatments, though flax and camelina SM amended treatments still contained significantly higher fungal populations compared with the control. The order of magnitude for fungal proliferation in the oilseed-amended microcosms was flax > camelina > jatropha.

Soil bacterial abundances were also increased following SM amendments compared with the control though to a smaller extent (four–sevenfold at day 14) than for fungi (Figure 1B). Thus, oilseed meal application resulted in significantly decreased soil bacterial to fungal ratios (75- to 150-fold at day 3) compared with the unamended treatment throughout the entire experiment (Figure 1C). The ratio tended to increase over time and then stabilized after day 21. The order of magnitude for bacterial proliferation in the oilseed-amended microcosms was flax > camelina > jatropha.

### Soil Fungal Community Composition

**FIGURE 2:**
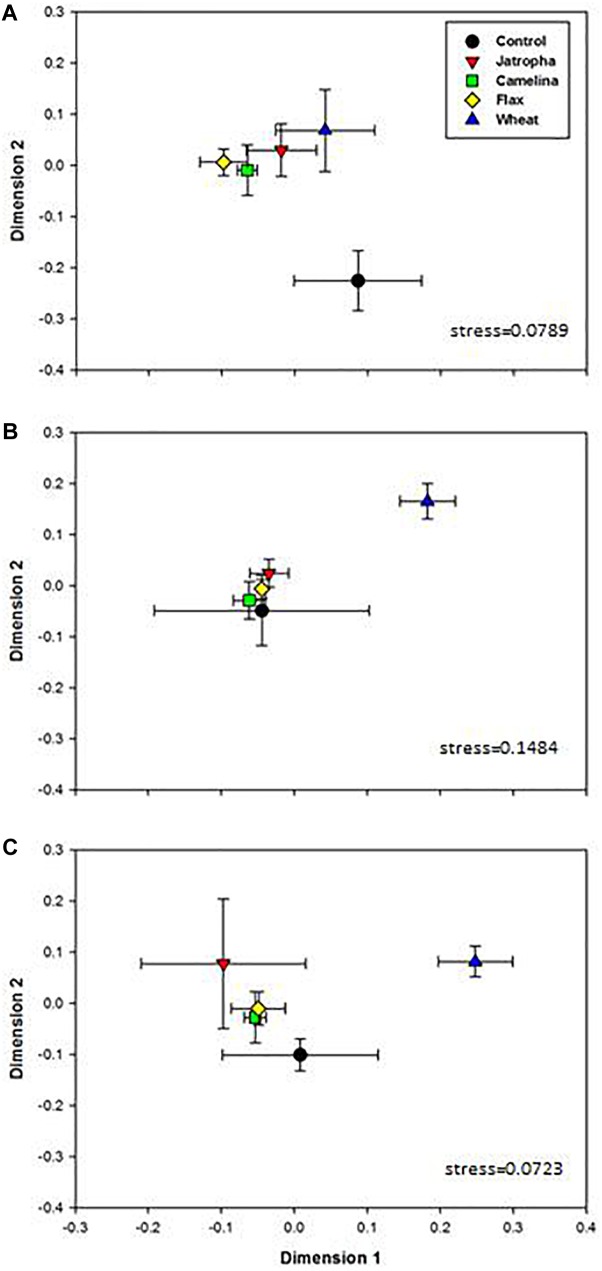
Non-metric multidimensional scaling (NMDS) ordination based on 1741 OTUs in 1% (w/w) organic material (SMs of jatropha, camelina, flax, and wheat straw) treated Weswood loam soil after 3 **(A)**, 21 **(B)**, and 77 **(C)** days of incubation at 25°C. Symbols represent the mean ordination of three biological replicates in each treatment, and the error bars represent the standard deviation among biological replicates. PERMANOVA was performed for each time point and *P*-values were shown.

Soil fungal community compositions based on OTU profiles were found significantly different with respect to amendment type and time of incubation, as indicated by two-way ANOSIM analysis (Supplementary Table S3). The NMDS analysis indicated that amendment of soil with the SMs of jatropha, camelina, and flax altered the soil fungal community composition (Figure 2). PERMANOVA indicated that within each time point, soil fungal structures were overall significantly different across amendments. At day 3 when soil fungal abundances had been promoted by SMs, community compositions in all three SM-amended treatments were significantly different from the unamended control (Figure 2A). However, by day 21 when both the soil fungal and bacterial populations stabilized, the soil fungal communities were very similar in all three SM treatments and the control (Figure 2B). In contrast, the wheat straw amendment also altered soil fungal community composition compared with the unamended control, but the shift was significantly different from that for the SMs and persisted throughout the 77 days of the experiment.

**FIGURE 3:**
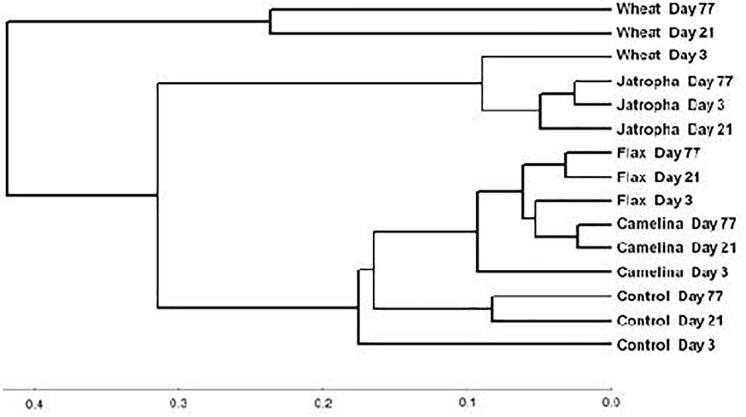
Neighbor joining tree based on Theta-YC similarity metrics for a Weswood loam soil amended with different organic amendments including SMs of jatropha, camelina, flax, and wheat straw as well as unamended control at sampling time points of 3, 21, and 77 days of incubation at 25°C. Biological replicates for each treatment were treated as one group to calculate the Theta-YC similarity metrics. Analysis was based on 1741 operational taxonomic units (OTUs) clustered at 97% sequence identities.

**FIGURE 4:**
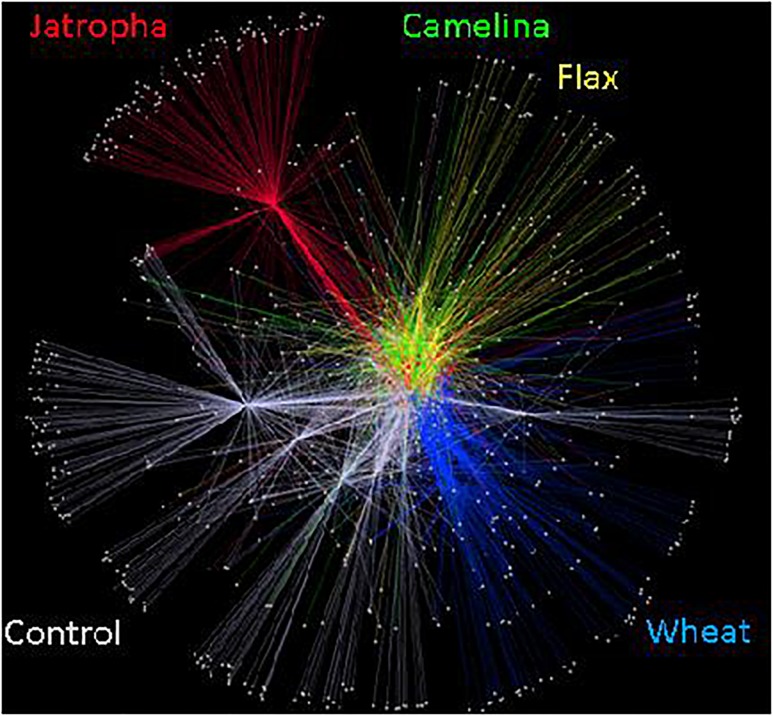
Soil fungal community OTU networks in Weswood loam soil amended with different organic amendments including SMs of jatropha, camelina, flax, and wheat straw as well as unamended control at sampling time points of 3, 21, and 77 days of incubation at 25°C. Analysis was conducted in Cytoscape 2.8.3 using embedded spring algorithm based on operational taxonomic units (OTUs) clustered at 97% sequence identities. Nodes represent OTUs that repelled each other, while edges represent the abundance of shared OTUs among samples. Different amendments at all incubation time have been grouped and represented by different colors.

The soil fungal community composition as described by Theta-YC similarity metrics also showed that regardless of time of incubation, all three SMs altered the fungal community composition (Figure 3). All fungal communities grouped by amendment type with camelina being most similar to flax, then the control, jatropha, and wheat amendments (in order of decreasing similarity). Within each treatment, the fungal communities from day 21 and day 77 were generally more similar to each other than the communities at day 3. One exception was the jatropha SM amended soils, where more similarity was shared between days 3 and 77 samples, though their differences from day 21 samples were relatively small. Community fungal OTU network across all time points showed overall differentiated fungal phylogenetic structure due to amendment type (Figure 4). Jatropha SM induced separated fungal structure from camelina and flax SMs, while all SM amendments resulted in different fungal community structure compared with wheat straw and unamended control.

**FIGURE 5:**
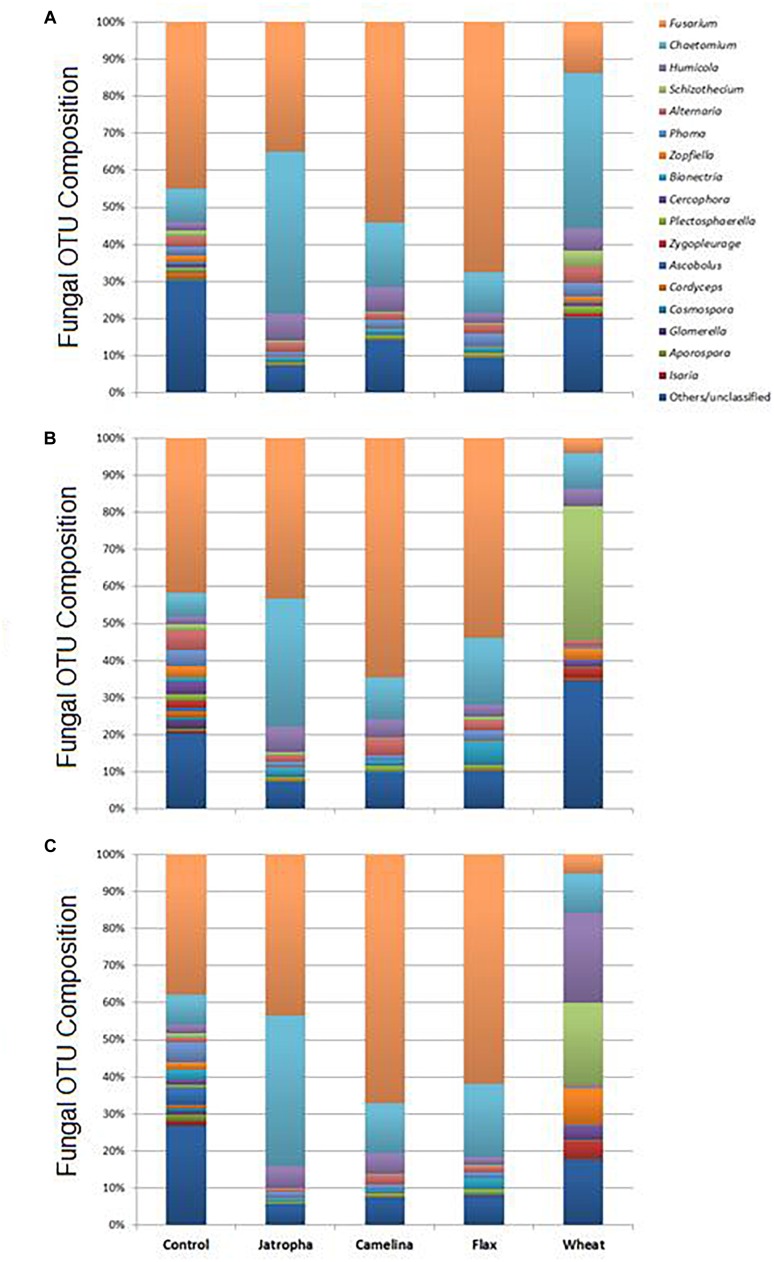
Fungal OTU distribution patterns summarized at the genus level in Weswood loam soils treated with SMs of jatropha, camelina, and flax, as well as wheat straw and unamended control after 3 **(A)**, 21 **(B)**, and 77 **(C)** days of incubation at 25°C. Bars represent the means of relative abundance of different OTUs of three biological replicates in each treatment at each time point.

As suggested by results from NMDS and Theta-YC, soil fungal taxonomic distribution patterns were also altered by SM applications, and these changes varied over time (Figure 5, Supplementary Table S4, and Supplementary Figure S2). All three SMs decreased soil fungal diversity compared with the unamended soil at all 3 time points (Supplementary Table S5). *Ascomycota* (>90%) was the dominant phylum of classified fungal groups in all treatments and the fungal genera shown in Supplementary Table S4 all belonged to this phylum. *Fusarium* and *Chaetomium* were the dominant genera detected in all SM treatments as well as the unamended control across time. Oilseed meals of camelina and flax significantly enhanced *Fusarium* than the unamended control. Jatropha SM showed very interesting result in consistently yielding lowest fungal diversity (Supplementary Table S5) and significantly higher *Chaetomium* composition compared with all the other SM treatments and the control throughout the incubation time (Supplementary Table S4).

### Soil Community Level Physiological Profiles (CLPP)

**FIGURE 6:**
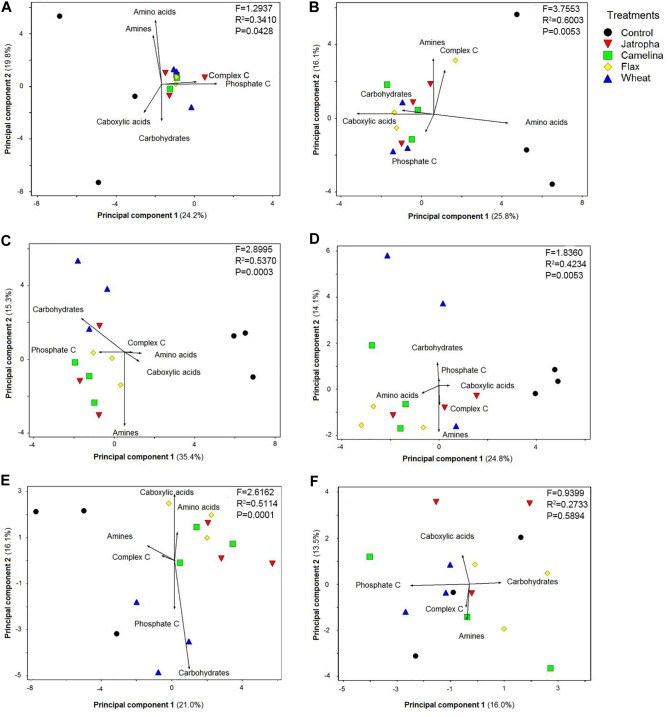
Principal component analysis (PCA) on Weswood loam soil microbial CLPP indicated by Biolog EcoPlate^TM^ with C sources grouped into carboxylic acids, complex C, carbohydrates, phosphate-containing C, amino acids, and amines after 3 **(A)**, 7 **(B)**, 14 **(C)**, 28 **(D)**, 77 **(E)**, and 133 **(F)** days of incubation at 25°C. Treatments included unamended control, 1.0% (w/w) SMs of jatropha, camelina, and flax, and wheat straw. Symbols represent mean values from each replicate-level sample. PERMANOVA was performed for each time point, *F-*, *R*^2^-, and *P*-values were shown.

Soil microbial functional patterns of CLPPs based upon C source utilization indicated by principal component analysis (PCA) were significantly affected by SM additions (Figure 6). All three SMs (jatropha, camelina, and flax) resulted in significantly different CLPPs from the control from day 3 through day 77 as indicated by PERMANOVA analysis. A PCA biplot was created to show how each of the six categories of C sources (Supplementary Table S1) contributed to the separation among treatments. The cumulative percentage of variation explained by principal components 1 and 2 ranged from 29.5 to 50.7%. At day 3, we found complex C and phosphate-associated C sources were the main contributors for the separation of organic amendments from the unamended control (Figure 6A). At day 7, the primary C sources contributing to CLPP separation were slightly different from day 3, with greater utilization of carboxylic acids and carbohydrates differentiating the organic amendments away from the unamended control (Figure 6B). By day 14, there was a differentiation between the SMs and wheat straw with the SMs observing greater utilization of phosphate-associated C and amines and the wheat straw having greater utilization of carbohydrates. This pattern continued with the wheat straw producing greater levels of utilization of carbohydrates and also phosphate-associated C at days 28 and 77. Likewise, the pattern continued for the SMs at days 28 and 77, with utilization of amines, amino acids, and then carboxylic acids being most positively impacted. At the end of the experiment (day 133), there was no substantial separation in CLPP among treatments (Figure 6F).

## Discussion

### Soil Fungal and Bacterial Abundance Suppressed by Jatropha and Camelina SMs

Compared with flax SM treatment that contain no biocidal chemicals, applying SM of jatropha that contain bioactive compounds including phorbol esters induced significantly overall lower fungal and bacterial proliferation, indicating jatropha SM have biofumigant features and worth further investigation. Recent studies demonstrated the antibacterial feature of jatropha on some bacterial pathogens (Okoh et al., 2016; Ha et al., 2018), and our data is the first report showing that jatropha SM can inhibit overall soil bacterial growth and has antifungal features as well. Treatment of camelina SM, which contains a moderate level of GLS analyzed in our previous study (Hu et al., 2011), also showed some evidence on biofumigation effect according to fungal abundance response. The fungal abundances peaked at day 3 in the flax treatment (no GLS) but did not peak until day 7 in the camelina treatment. This delayed peak in fungal abundances in the camelina SM treatment may have resulted from the release of fungicidal chemicals such as ITCs from the GLS. Similar trends were found in previous studies of Hollister et al. (2013) using mustard (contained GLS) SM amended soil and in Hu et al. (2015) using flax SM with addition of allyl ITC. Our results suggested that relatively low concentrations of GLS-induced allelochemicals from camelina could only reduce the overall soil fungal abundance temporarily without selectivity. ITC related suppression on an overall fungal community without selectivity was also reported in a previous research (Hu et al., 2015). Other studies have reported that such chemicals do not always produce negative impacts on soil total fungal abundance (Wang et al., 2012). However, this appeared to be due to selection and proliferation of a small number of specific fungal groups that were apparently more resistant to ITCs (Hollister et al., 2013; Hu et al., 2015).

Soil fungal abundances increased (∼40-fold) much more than bacterial abundances (4–7-fold) by addition of the SMs, suggesting that soil fungi were more responsible for the decomposition of added SMs than were soil bacteria. The lab incubation under conditions in our study may have been biased toward fungi, while different soil temp or water content may also have favored other organisms. Similar results were reported in a previous study comparing SMs of *Brassica juncea* amendments with unamended soil (Hollister et al., 2013). Soil bacterial abundances in the oilseed amended treatments peaked at day 14, after soil fungal abundances had decreased. This delayed response may have resulted from bacteria feeding on living or dead fungal hyphae (Boer et al., 2010), or because of less competition for resources from the largely decreased number of fungi.

### Soil Fungal Community Structural Shifts by Jatropha and Camelina SMs

In our study, although the differences in the composition of the soil fungal communities among the three SM amendments was not apparent indicated by NMDS, we still found a trend that fungal communities were clustered by SM type, especially jatropha versus camelina and flax. Since the above three SMs have comparable nutrient compositions, such variation in fungal community structure indicate additional features jatropha SM has that induced differentiated microbial responses.

Another explanation for changes in soil fungal community composition by SM application could be attributed to biofumigation effects by allelochemicals released from selected SMs (Hollister et al., 2013; Mazzola et al., 2014; Yim et al., 2017). Jatropha SM that containing bioactive compounds induced significantly lower overall fungal biodiversity and higher *Chaetomium* composition compared with other 2 SM amendments, indicating the bioactive compounds in jatropha SM can change soil fungal community differently than widely studied *Brassicaceae* family. Oilseed meals from *Brassicaceae* family such as *B. juncea* (Indian mustard) was reported to increase relative abundance of *Retroconis* (Hollister et al., 2013), but in our study we found generally no fumigant impact of camelina SM throughout time. This could be related to the lower level of GLS in camelina compared with *B. juncea* and/or the type of GLS (Hu et al., 2015). Similar as our study, low application rate of brassicaceous SM has been previously reported having no significant impact on soil general fungal and bacterial structures as well (Wei et al., 2016). For the targeted pathogens, camelina SM was also found not suppressive to *Fusarium* wilt, while *Brassica juncea* SM containing higher level of GLS was (Ren et al., 2018). In the present study, the only result that may suggest allelochemical impacts on soil fungal composition in camelina SM amendment would be its relatively higher dissimilarity at day 3 compared with flax SM treatment.

One more explanation for shifts in the soil fungal community composition could be attributed to soil bacterial community responses and the interactions between the fungal and bacterial populations. Evidence for strong bacterial–fungal antagonism in global topsoil has been provided in a previous research (Bahram et al., 2018). And previous studies consistently showed that the incorporation of specific SMs could increase fungal antagonists such as *Bacillus*, *Pseudomonas*, and *Streptomyces* spp. (Hollister et al., 2013; Ren et al., 2018), and significantly decreased *Chloroflexi*, which was suspected to be involved in fungal pathogen suppression (Ren et al., 2018). Hu et al. (2015) also reported an increased fungal antagonist *Paenibacillus* composition following ITC amended SM addition. Although we did not directly analyze soil bacterial community compositions or antibiotic-resistance genes to reveal the presence of antagonism, we did find significantly decreased bacterial to fungal ratios across all SM treatments, indicating antagonism between bacteria and fungi competing for space and food.

Camelina SM that are rich in N content mainly promoted *Fusarium* spp., which was also the dominant genus in SM amended and unamended soil. Such increase of *Fusarium* in our study may not be detrimental to the ecosystem since most *Fusarium* species in soil are non-pathogenic and harmless to the environment. In addition, the soil in our study did not have a history of *Fusarium*-caused disease in the past. Such increased *Fusarium* could be beneficial for agriculture since non-pathogenic *Fusarium* has been found economically and ecologically important and positively correlated to pathogenic *Fusarium* suppression in support of the competition theory for nutrients between non-pathogenic and pathogenic *Fusarium* (Scher and Baker, 1982; Lemanceau et al., 1988).

Differences in the fungal composition in the jatropha amended soil were likely to be mostly due to the high percentages of *Chaetomium*, some members of which have been used in biotechnological industry due to their high selectivity for assimilating polysaccharides, especially hemicelluloses such as xylan, as well as their ability to produce enzymes such as cellulase and laccase (Ankudimova et al., 1999; Mimura et al., 1999; Suyanto et al., 2003). Research by Suyanto et al. (2003) indicates the potential of particular *Chaetomium* in decomposition of palm-oil mill fiber, which is somewhat similar to our kernel-containing jatropha SM. Other *Chaetomium* have been demonstrated to assist biological control of particular pathogens through production of toxic metabolites and/or competition for living space and nutrient resources (Aggarwall et al., 2004; Zhang and Yang, 2007; Kharwar et al., 2010). Other Ascomycota members including *Aspergillus awamori*, *Aspergillus nidulans*, and *Trichoderma viride* inoculants have also been reported effectively decompose jatropha de-oiled cake (Chaturvedi et al., 2013), suggesting the importance of studying soil fungi that are resistant to jatropha induced toxic chemicals.

### Soil Microbial Functional Changes

No major differences among jatropha, camelina, and flax SM amendments were detected in our study. Although utilization of C sources in the Biolog EcoPlates do not necessarily represent *in situ* degradation of the C substrates in the microcosms, changes in the utilization patterns over time do indicate shifts in the capacity of the microbial communities to metabolize different C sources (Gomez et al., 2006). Taking along with varied microbial abundance and structure among the SMs in this study, the CLPP patterns suggested relatively stable and similar microbial function in nutrient utilization regardless of allelochemicals SM released.

The PCA biplots of the CLPP results indicated a temporal trend of shifts in microbial degradation of various types of organic materials according to nutrient compositions. At day 3, soil microorganisms in SM-applied microcosms tended to utilize complex C and phosphate-associated C than the control. This trend shifted to carbohydrates and carboxylic acids by day 7 and then amines and amino acids at days 14–77. The apparent sequential hierarchy for C metabolism in the oilseed-amended microcosms was generally complex C > phosphate-associated C > carboxylic acids > carbohydrates > amines > amino acids. This suggests that the residual oils were degraded first followed by P-containing compounds, then the other C-containing compounds, and lastly the N-containing compounds. This is the first report on CLPP pattern of microbial community changes after SM incorporation to soil. Thus there are no previous studies to compare with our results on CLPP. A previous study reported that brassicaceous SMs did not change soil biodiversity based on Biolog analysis (Scotti et al., 2018), but no CLPP pattern was summarized and reported.

### Soil Microbial Community Succession

Soil microbial communities can progress through successional stages during ecosystem development (Nemergut et al., 2007; Schütte et al., 2009; Brown and Jumpponen, 2013), and fertilization alone has been demonstrated influencing soil microbial community development and accelerating succession rate (Knelman et al., 2014). In our study, the dynamics of microbial community changes though time of incubation could partially come from addition of nutrients and biocidal compounds from SMs, and partially resulted from natural microbial succession or random processes.

Overall, fungal community structures across all microcosms except jatropha SM amendments were shifted from the beginning of the experiment (day 3) to middle stage of incubation (day 21), and stabilized afterwards. This trend in unamended control implied a natural microbial succession existed independent of treatment. If more time points before day 21 when stabilized were tested on fungal structures, differentiated succession due to SM amendments might be revealed. Fungal population abundance changes due to succession through time were negligible in this study. In the unamended control, soil bacterial abundances gradually increased and peaked after 2–3 weeks of incubation, then decreased and stabilized. Oilseed meal amendments on the other hand resulted in bacterial abundance peak a little earlier (day 14), indicating possible accelerated succession rate due to addition of nutrients (Knelman et al., 2014).

### Novelties and Limitations

This is the first report on soil fungal responses to dedicated biofuel crop jatropha and camelina SM amendments, from the perspectives of absolute abundance and sequence-based community structural changes. We are also the first to investigate temporal microbial functional shifts after SM amendments indicated by CLPP using Biolog EcoPlates. Our study provided novel information but also has limitations. This is a lab based microcosm study with limited space and artificial conditions. Since soil microbial communities can be affected by a number of environmental factors, further research should be conducted in greenhouse and in the field conditions to investigate the impact of applying selected SMs on soil microbiota in real practice.

## Conclusion

The different SM amendments seemed to result in slightly varied soil microbial community responses depending on either the allelochemicals involved, nutrient composition (e.g., C:N ratio), or both. Application of jatropha and camelina SMs to soil rapidly increased microbial, especially fungal, abundances compared with unamended control. Jatropha SM showed significant biofumigation effect on soil fungi with suppressed fungal abundance and shifted fungal community structure compared with camelina and flax SM amendments. Jatropha SM promoted *Chaetomium*, while camelina SM enhanced *Fusarium* proliferation. Regardless of SM type, there was a sequential hierarchy for C metabolism in SM amended microcosm, where the residual oils were degraded first followed by P-containing compounds, then the other C-containing compounds, and lastly the N-containing compounds. Our study revealed biofumigant and nutritional influence of SMs from dedicated biofuel plants on soil microbial communities. This information will help properly using jatropha and camelina SMs for pathogen control while minimizing their negative impacts on non-target microorganisms. However, further studies in the field are demanded to investigate their influences in real practice.

## Data Availability

Publicly available datasets were analyzed in this study. This data can be found here: https://www.ncbi.nlm.nih.gov/Traces/study/?acc=PRJNA513249.

## Author Contributions

PH led the experimental design, carried out the experiments, performed data analysis, and wrote the manuscript. LW contributed to bioinformatics analysis and writing. EH and AS contributed to sequencing data interpretation. AW contributed experiments and data analysis on soil mineralization rate. FH contributed to the experimental design. TG is the supervisor of the project and supported the research.

## Conflict of Interest Statement

The authors declare that the research was conducted in the absence of any commercial or financial relationships that could be construed as a potential conflict of interest.
