# Supplementary material for: Fungal Community Structural and Microbial Functional Pattern Changes After Soil Amendments by Oilseed Meals of Jatropha curcas and Camelina sativa: A Microcosm Study
Source: Front Microbiol. 2019 Mar 29;10:537. doi: 10.3389/fmicb.2019.00537 (PMC6450180; doi:10.3389/fmicb.2019.00537)
Supplement: Supplementary file 1 [file Data_Sheet_1.docx]

Table S1. Carbon (C) groupings of 31 C sources used in Biolog EcoPlate™.

| **C group*** | **C source** | **Well location** |
| --- | --- | --- |
| Carboxylic acids | Pyruvic acid methyl ester | B1 |
|  | D-glucosamic acid γ-lactone | F2 |
|  | D-galactonic acid | A3 |
|  | D-galacturonic acid | B3 |
|  | 2-hydroxy benzoic acid | C3 |
|  | 4-hydroxy benzoic acid | D3 |
|  | γ-hydroxy butyric acid | E3 |
|  | Itaconic acid | F3 |
|  | α-ketobutyric acid | G3 |
|  | D-malic acid | H3 |
| Complex C sources | Tween 40 | C1 |
|  | Tween 80 | D1 |
|  | α-cyclodextrin | E1 |
|  | Glycogen | F1 |
| Carbohydrates | D-cellobiose | G1 |
|  | α-D-lactose | H1 |
|  | β-methyl-D-glucoside | A2 |
|  | D-xylose | B2 |
|  | i-erythritol | C2 |
|  | D-mannitol | D2 |
|  | N-acetyl-D-glucosamine | E2 |
| Phosphate C | Glucose-1-phosphate | G2 |
|  | D,1-α-glycerol phosphate | H2 |
| Amino Acids | L-arginine | A4 |
|  | L-asparagine | B4 |
|  | 1-phenylalanine | C4 |
|  | L-serine | D4 |
|  | L-threonine | E4 |
|  | Glycyl-L-glutamic acid | F4 |
| Amines | Phenylethyl-amine | G4 |
|  | Putrescine | H4 |

*Adapted from Chazarenc et al., 2010.

Table S2. Selected elemental concentrations of SMs of jatropha, camelina, flax, and wheat straw.*

| Biomass | C | N | P | K | Ca | S | Mg | Na | Zn | Fe | Cu | Mn | B |
| --- | --- | --- | --- | --- | --- | --- | --- | --- | --- | --- | --- | --- | --- |
| Type | --------------------------- g kg^-1^ ----------------------- | | | | | | | | ----------- mg kg^-1^ ------- | | | | |
| Jatropha | 477 | 35 | 6.2 | 12.9 | 9.6 | 2.3 | 5.2 | 1.1 | 31 | 42 | 21 | 39 | 30 |
| Camelina | 439 | 59 | 10.1 | 14.5 | 5.3 | 12.3 | 4.2 | 0.4 | 71 | 53 | 12 | 42 | 24 |
| Flax | 491 | 51 | 6.8 | 10.3 | 3.0 | 3.3 | 5.1 | 0.7 | 38 | 33 | 13 | 34 | 27 |
| Wheat | 416 | 13 | 1.1 | 16.0 | 2.6 | 1.1 | 1.0 | 1.3 | 15 | 194 | 7 | 53 | <9 |

(* Modified from Hu et al., 2011)

Table S3. Analysis of similarity based on OTU composition with respect to SM and wheat straw amendment and time.

| Microbial community | Treatment | ANOSIM metric | |
| --- | --- | --- | --- |
|  |  | *R* | *P* value |
| Fungi | Amendment | 0.5556 | <0.0001 |
|  | Time | 0.2543 | 0.004 |

Table S4. Fungal OTU composition at the genus level in Weswood loam soils treated with SMs of jatropha, camelina, and flax, as well as wheat straw and unamended control after 3, 21, and 77 days of incubation at 25°C. Values represent the means of 3 biological replicates in each treatment.

| **Genus** | **Control** | **Jatropha** | **Camelina** | **Flax** | **Wheat** | **Control** | **Jatropha** | **Camelina** | **Flax** | **Wheat** | **Control** | **Jatropha** | **Camelina** | **Flax** | **Wheat** |
| --- | --- | --- | --- | --- | --- | --- | --- | --- | --- | --- | --- | --- | --- | --- | --- |
|  | **Fungal OTU Composition %** | | | | | | | | | | | | | | |
|  | **Day 3** | | | | | **Day 21** | | | | | **Day 77** | | | | |
| *Fusarium* | 45.07b* | 34.94b | 54.19ab | 67.53a | 13.82c | 41.85c | 43.24c | 64.59a | 53.85b | 4.07d | 37.95b | 43.53b | 67.03a | 61.95a | 5.07c |
| *Chaetomium* | 8.76b | 43.67a | 17.33ab | 10.78b | 41.86a | 6.26b | 34.57a | 11.23b | 17.93b | 9.43b | 7.97b | 40.54a | 13.54b | 19.60b | 10.73b |
| *Schizothecium* | 1.81b | 0.44b | 0.32b | 0.36b | 4.21a | 1.82b | 0.76b | 0.22b | 0.96b | 36.20a | 1.31b | 0.17c | 0.21c | 0.53bc | 22.15a |
| *Humicola* | 2.34b | 7.47a | 6.75a | 2.98ab | 6.05a | 2.00b | 6.94a | 4.92ab | 3.39ab | 4.89ab | 2.26c | 6.06b | 5.64b | 2.20c | 24.12a |
| *Alternaria* | 2.59ab | 2.51ab | 1.81b | 2.32ab | 4.53a | 5.28a | 1.77bc | 4.68ab | 2.79abc | 1.53c | 1.11bc | 0.65cd | 2.55a | 1.48ab | 0.35d |
| *Phoma* | 2.44a | 1.32a | 2.42a | 3.46a | 3.53a | 4.25a | 1.48bc | 1.19cd | 2.69ab | 0.51d | 5.43a | 1.75b | 1.60b | 1.57b | 0.64b |
| *Zopfiella* | 1.79a | 0.28b | 0.12b | 0.25b | 1.56a | 3.01a | 0.25b | 0.07b | 0.16b | 3.11a | 2.05b | 0.28bc | 0.03c | 0.03c | 9.99a |
| *Bionectria* | 0.41ab | 1.01ab | 1.35a | 1.39a | 0.20b | 1.36b | 2.21b | 1.30b | 6.10a | 0.19c | 2.70ab | 0.88bc | 0.59bc | 2.99a | 0.09c |
| *Cercophora* | 0.96a | 0.27a | 0.13a | 0.15a | 0.80a | 3.15a | 0.12b | 0.07b | 0.17b | 1.70a | 1.27b | 0.01c | 0.10bc | 0.05bc | 3.85a |
| *Plectosphaerella* | 1.10a | 0.31a | 0.87a | 0.99a | 2.07a | 1.88a | 0.72ab | 1.70ab | 1.20ab | 0.35b | 0.84a | 0.43a | 0.91a | 1.17a | 0.32a |
| *Zygopleurage* | 0.00c | 0.01bc | 0.05b | 0.05bc | 0.74a | 1.83a | 0.04b | 0.00b | 0.01b | 2.67a | 0.13b | 0.00b | 0.04b | 0.00b | 4.78a |
| *Ascobolus* | 0.17a | 0.01b | 0.01b | 0.01b | 0.01b | 1.03a | 0.00a | 0.05a | 0.01a | 0.02a | 4.50a | 0.10a | 0.04a | 0.66a | 0.01a |
| *Cordyceps* | 1.95a | 0.22a | 0.18a | 0.27a | 0.31a | 1.69a | 0.28b | 0.16b | 0.30b | 0.42b | 0.87a | 0.03b | 0.12b | 0.05b | 0.06b |
| *Cosmospora* | 0.78a | 0.11b | 0.11b | 0.17b | 0.12b | 0.85a | 0.08bc | 0.27b | 0.26b | 0.05c | 0.80a | 0.01b | 0.09b | 0.07b | 0.02b |
| *Glomerella* | 0.05a | 0.00a | 0.01a | 0.01a | 0.01a | 2.09a | 0.01b | 0.01b | 0.00b | 0.02b | 0.94a | 0.00b | 0.00b | 0.01b | 0.00b |
| *Aporospora* | 0.00c | 0.05b | 0.10ab | 0.05b | 0.16a | 0.77a | 0.18ab | 0.04b | 0.06b | 0.05b | 1.80a | 0.03b | 0.07b | 0.01b | 0.08b |
| *Isaria* | 0.00c | 0.00b | 0.00bc | 0.00c | 0.02a | 0.66a | 0.02ab | 0.02b | 0.01b | 0.06b | 1.33a | 0.02b | 0.00b | 0.02b | 0.31b |
| Others  /unclassified | 29.78 | 7.37 | 14.26 | 9.24 | 20.02 | 20.22 | 7.34 | 9.47 | 10.10 | 34.72 | 26.72 | 5.51 | 7.44 | 7.62 | 17.42 |

*Different letters indicate significant difference within each row at a specific time point.

Table S5. Summary of fungal community characteristics based on OTUs (97% similarity) and their relative abundances in a Weswood loam soil amended with 1.0% (w/w) oilseed meals of jatropha, camelina, flax, and wheat straw after 3, 21, and 77 days of incubation at 25 °C. Diversity and richness estimates are based upon normalized (reduced-sized) sequence libraries, each of which contained 730 sequences. Values represented for the means of 3 biological replicates with standard deviations.

| Sample | | Community characteristics (mean ± std) | | | |
| --- | --- | --- | --- | --- | --- |
| Amendment | Day | Observed OTUs | Chao I richness | Shannon (H’) | Inverse Simpson |
| Control | 3 | 67±18 | 82±31 | 2.90±0.65 | 12.0±9.3 |
| Jatropha | 3 | 51±3 | 83±15 | 2.12±0.13 | 4.3±0.8 |
| Camelina | 3 | 55±6 | 88±15 | 2.55±0.17 | 7.6±1.0 |
| Flax | 3 | 59±8 | 94±8 | 2.47±0.12 | 6.7±1.0 |
| Wheat | 3 | 73±7 | 147±69 | 2.55±0.46 | 6.3±4.9 |
| Control | 21 | 103±10 | 176±54 | 3.41±0.15 | 14.5±2.4 |
| Jatropha | 21 | 64±12 | 107±42 | 2.33±0.07 | 5.4±0.6 |
| Camelina | 21 | 62±14 | 94±29 | 2.48±0.17 | 6.7±0.5 |
| Flax | 21 | 66±8 | 123±17 | 2.57±0.11 | 7.3±0.4 |
| Wheat | 21 | 75±24 | 157±33 | 2.46±0.34 | 5.1±0.6 |
| Control | 77 | 92±13 | 138±34 | 3.38±0.36 | 17.0±6.2 |
| Jatropha | 77 | 48±10 | 123±66 | 1.93±0.28 | 4.1±1.1 |
| Camelina | 77 | 60±9 | 134±55 | 2.32±0.13 | 5.9±0.4 |
| Flax | 77 | 56±15 | 103±23 | 2.32±0.25 | 6.2±1.1 |
| Wheat | 77 | 61±2 | 87±7 | 2.68±0.09 | 7.8±0.2 |

Figure S1. Soil C mineralization dynamics as affected by added oilseed meals and wheat biomass over time. (●: control; ○: 0.5%; ▼: 1%; Δ: 5% application rate). Means are based on 3 replications. Bars represent ± standard deviation of the mean. Error bars are hidden when smaller than the symbols.

Figure S2. Heatmap on soil fungal communities genus level in Weswood loam soils treated with oilseed meals of jatropha, camelina, and flax, as well as wheat straw and unamended control after 3, 21, and 77 days of incubation at 25°C. Abundances for each taxonomic group were scaled relative to the mean across all samples. The dendrogram depicts Euclidian distance-based hierarchical agglomerative clustering of samples with one another.
